# Unlocking the Heterogeneity in Acute Leukaemia: Dissection of Clonal Architecture and Metabolic Properties for Clinical Interventions

**DOI:** 10.3390/ijms26010045

**Published:** 2024-12-24

**Authors:** Martina Maria Capelletti, Orsola Montini, Emilio Ruini, Sarah Tettamanti, Angela Maria Savino, Jolanda Sarno

**Affiliations:** 1 School of Medicine and Surgery, University of Milan-Bicocca, 20126 Milan, Italy; martina.capelletti@unimib.it (M.M.C.); o.montini@campus.unimib.it (O.M.); ruiniemilio@gmail.com (E.R.); angela.savino@unimib.it (A.M.S.); 2 Tettamanti Center, Fondazione IRCCS San Gerardo dei Tintori, 20900 Monza, Italy

**Keywords:** intratumour heterogeneity, metabolism, single-cell technologies, acute leukaemia

## Abstract

Genetic studies of haematological cancers have pointed out the heterogeneity of leukaemia in its different subpopulations, with distinct mutations and characteristics, impacting the treatment response. Next-generation sequencing (NGS) and genome-wide analyses, as well as single-cell technologies, have offered unprecedented insights into the clonal heterogeneity within the same tumour. A key component of this heterogeneity that remains unexplored is the intracellular metabolome, a dynamic network that determines cell functions, signalling, epigenome regulation, immunity and inflammation. Understanding the metabolic diversities among cancer cells and their surrounding environments is therefore essential in unravelling the complexities of leukaemia and improving therapeutic strategies. Here, we describe the currently available methodologies and approaches to addressing the dynamic heterogeneity of leukaemia progression. In the second section, we focus on metabolic leukaemic vulnerabilities in acute myeloid leukaemia (AML) and acute lymphoblastic leukaemia (ALL). Lastly, we provide a comprehensive overview of the most interesting clinical trials designed to target these metabolic dependencies, highlighting their potential to advance therapeutic strategies in leukaemia treatment. The integration of multi-omics data for cancer identification with the metabolic states of tumour cells will enable a comprehensive “micro-to-macro” approach for the refinement of clinical practices and delivery of personalised therapies.

## 1. Introduction

Tumours are composed of cells of various types, in different states and with different functions. Untangling the interpatient and intratumour heterogeneity in haematological diseases is crucial to target the cellular populations responsible for resistance to therapy and relapse. In leukaemia, different subpopulations with distinct mutations and characteristics arise and coexist in the same niche in the form of subclones. The presence of these heterogeneous subclones, as the disease evolves, leads to genetic diversity within the tumour, impacting the response to treatment and drug resistance. The introduction of next-generation sequencing (NGS) technologies has drastically expanded our understanding of the clonal heterogeneity leading to inter- and intrapatient diversity. Genome-wide analyses performed on thousands of patients with acute lymphoblastic leukaemia (ALL) and acute myeloid leukaemia (AML) have led to the identification of more than 50 different subtypes, either genetically or transcriptionally driven [1,2]. Moreover, the widespread nature of single-cell technologies has allowed researchers to gain unprecedented insights into clonal heterogeneity by revealing the extent of variation between individual cancer cells within the same tumour. Despite these advances, one critical and largely unexplored component of tumour heterogeneity is the intracellular metabolome, a repertoire of cellular metabolites and lipids, guiding cell-autonomous and non-autonomous processes. AML and ALL are both aggressive forms of blood cancer that originate in the bone marrow and rapidly affect healthy haematopoiesis. Recent studies have shown how AML and ALL exhibit altered metabolic pathways, supporting the rapid growth and proliferation of malignant cells [3]. In leukaemia, the abnormal metabolism often includes increased glucose uptake, altered mitochondrial function, and dysregulated biosynthetic pathways that help to fuel the synthesis of cellular components necessary for cell division [4,5]. Distinct leukaemic cell subpopulations exhibit a reliance on specific metabolic pathways, which are influenced by their genetic mutations, the immune microenvironment and the availability of nutrients within the bone marrow niche. Understanding how these metabolic shifts contribute to leukemogenesis and resistance to treatment could open up potential therapeutic avenues that target the metabolic vulnerabilities of leukaemia cells, offering new strategies for treatment [6].

Over the past few years, the development of single-cell metabolomics methods, initially pioneered by research in yeast cells [7,8], has demonstrated the feasibility of characterising metabolic heterogeneity and discriminating between cell types [9,10,11,12,13]. Single-cell technologies play a critical role in advancing leukaemia treatment, enabling the more precise identification of resistant profiles of single cells and effective therapeutic strategies to target them. Introducing these technologies for the study of the mechanism of resistance in the context of leukaemia treatment is crucial to achieve a significant impact in terms of better patient outcomes.

In this review, we aim to bridge the gap between understanding metabolic heterogeneity in leukaemia and its potential therapeutic implications. Acute leukaemia, being a highly aggressive and treatment-resistant malignancy, thrives in the bone marrow niche, a unique microenvironment that plays a pivotal role in supporting leukaemic cell survival, proliferation and resistance to therapy. The bone marrow niche not only provides a sanctuary for leukaemic cells but also shapes their metabolic adaptations through interactions with stromal cells and immune cells and the availability of nutrients [14,15]. By consolidating and analysing recent advancements in single-cell technologies and metabolomics, this review sheds light on the intricate metabolic adaptations and vulnerabilities of leukaemic cells that drive disease progression, therapeutic resistance and relapse. Moreover, this review emphasises the potential to exploit these vulnerabilities to develop targeted and personalised treatments, which are urgently needed to overcome the limitations of conventional therapies. Highlighting these aspects is crucial in guiding future research and informing clinical strategies aimed to improve patient outcomes in acute leukaemia.

## 2. Experimental Approaches to Unravel the Genetic and Dynamic Heterogeneity of Leukaemic Cells

This section explores cutting-edge methodologies and their contributions to understanding the intricate landscape of leukaemic cell heterogeneity, focusing on genetics, transcriptomics, proteomics and metabolomics.

### 2.1. Genetics—scDNA-Seq

The field of leukaemia genomics has evolved significantly, from bulk sequencing methods like whole-exome sequencing (WES) to more refined single-cell techniques such as single-cell DNA sequencing (scDNA-Seq). Although WES is now more available and affordable, this analysis can only provide an average snapshot of the mutations present in a patient sample, masking the genetic intratumour heterogeneity. In an attempt to interrogate somatic mutations at a single-cell level, several scDNA-Seq technologies have been developed in the last decade [16]. In the field of leukaemia, Tapestri represents one of the most promising technologies to identify rare subclones, as well as resolving the clonal architecture, monitoring disease progression and potentially predicting treatment resistance [17]. Tapestri, from Mission Bio Inc., is a high-throughput scDNA-Seq technology that allows for the simultaneous measurement of DNA and proteins in thousands of individual cells. It can detect single-nucleotide variations (SNV), indels, copy number variations (CNV) and protein expression using oligonucleotide-conjugated antibodies designed for use in single-cell proteogenomics (i.e., Total-Seq D) [18]. Cells—about a million for each sample—are first stained with antibodies targeting surface antigens. A pre-built Total-Seq D Heme/Oncology panel with 45 antibodies is also available for this purpose [19]. Once stained, the cells are introduced into a microfluidic platform to encapsulate single cells in droplets, where unique barcodes label their DNA and proteins. The droplets are then broken, the barcoded material is amplified, and a library is prepared for sequencing to analyse each cell individually. Tapestri offers several advantages over earlier scDNA-Seq methods, such as the detection of low-frequency mutations, with a limit of detection of 0.01% cells. In addition, using a targeted sequencing approach increases the depth of coverage, improving the accuracy in detecting mutations at single-cell level. A portfolio of pre-designed panels for haematological diseases (AML and ALL) is available. Additionally, custom panels can be created to target specific genes of interest, allowing for greater flexibility and precision in genetic analyses. The main challenge to the broader applicability of the technology is the cost of the assay.

This will depend on several factors: the library preparation kit (DNA only vs. DNA + proteins); the DNA panel (pre-built vs. custom), understood as the number of amplicons needed; the antibody panel using Total-Seq D antibodies; and the sequencing depth. Consequently, the sequencing depth depends on the number of cells captured (~5000 cells with the first generation of Tapestri; 11,000 cells with the latest version, V3), the number of amplicons used in the DNA panel (80× read pairs per amplicon) and the number of oligo-conjugated antibodies used (1000× read pairs per antibody). Therefore, the total number of reads needed for the single-cell analysis of DNA mutations and protein expression will be calculated using the following formula: (number of cells × number of amplicons × 80) + (number of cells × number of antibodies × 1000). The sequencing, and consequently the assay cost for each sample, will vary depending on the required number of reads. To analyse the data, several tools are available for variant calling (i.e., InferCNV, Monovar), phylogenetic and clonality analysis (i.e., SciClone, CloneAlign) and the integrated analysis of DNA and proteins at a single-cell level (i.e., Mosaic, Optima).

### 2.2. Transcriptomics—scRNASeq

Single-cell RNA-Seq (scRNA-Seq) technology provides a high-resolution outlook on the transcriptional states of thousands of individual cells, capturing their gene expression profiles. This method has been instrumental in studying diverse biological processes, including developmental biology, tissue heterogeneity and cancer disease states. Functional studies have shown how even genetically similar cells can show significant functional diversity, with differences in their proliferative states, metabolic activity or immune evasion mechanisms [20,21]. These functional subpopulations can correlate with specific genetic clones, linking specific mutations to unique transcriptional programs [22]. While scRNA-Seq is focused on transcriptomics, it can also be used to infer clonal relationships through the identification of certain mutations within the transcribed RNA. More recently, a more advanced version of scRNA-Seq has been developed to detect both transcript and protein expression, named Cellular Indexing of Transcriptomes and Epitopes by Sequencing (CITE-seq) [23]. Several companies offer different solutions for single-cell capture and sequencing: from plate-based approaches where each well contains reagents for reverse transcription and libraries (i.e., SMART-Seq2, MARS-Seq) [24,25] to microwell (i.e., Rhapsody) [26] or microfluidics-based methods where cells are captured into droplets or wells (10× Chromium, Fluidgm C1) [27]. Sequencing can be either targeted or untargeted, affecting the depth of the coverage and the cost of the assay. Several pipelines for analysis, developed either in R or Python (i.e., Seurat, Scanpy) [28,29], are available to analyse and integrate scRNA-Seq data with other multi-omics approaches. In the context of leukaemia, the use of scRNA-Seq has been instrumental in better characterising AML leukaemia stem cells (LSCs) [30] and identifying transcriptional programs associated with relapse in ALL [20]. Furthermore, scRNA-Seq methods have been also employed to dissect intrinsic cellular differentiation patterns and identify different leukaemia subgroups in both AML [31] and ALL patients [32]. More recently, scRNA-Seq has also been combined with spatial transcriptomics to interrogate the tumour microenvironment composition within the bone marrow niche. This approach provides similar insights to scRNA-Seq, while preserving the spatial resolution of the sample, allowing for a more precise understanding of how leukaemia cells interact with their surrounding microenvironment in situ [33]. In AML, spatial transcriptomics has been used to examine proliferative phenotypes in the bone marrow niche, identifying DDP4 as a new marker for therapeutic intervention, which was detected only when performing in vivo imaging analysis [33]. Additionally, spatial transcriptomics revealed how T-cells are dysfunctional in AML, revealing unique immune landscapes associated with TP53 mutations [34]. These findings emphasise how spatially resolved data can uncover niche-specific gene expression and immune interactions, potentially guiding targeted therapies against the tumour–microenvironment crosstalk.

### 2.3. Proteomics—CyTOF and Mass Spectrometry

Two advanced technologies, cytometry by time-of-flight (CyTOF) and mass spectrometry (MS), are widely used to profile the proteome in leukaemia. These techniques provide high-dimensional and quantitative data on protein expression and post-translational modifications, giving valuable insights into the heterogeneity of leukaemia cells and the tumour microenvironment.

#### 2.3.1. Cytometry by Time-of-Flight (CyTOF)

Mass cytometry has significantly transformed the possibility of performing high-dimensional immunophenotyping, allowing researchers to analyse a larger number of parameters simultaneously. This technology combines mass spectrometry with flow cytometry, replacing fluorescently labelled antibodies with metal-conjugated antibodies, thereby overcoming challenges associated with spectral overlap and cellular auto-fluorescence [35]. Single cells are vaporised, and the metal tags are ionised and analysed by a time-of-flight mass spectrometer, which separates and quantifies the ions based on their mass [35]. High-dimensional data for individual cells are generated, revealing protein expression profiles that help to identify distinct subclonal populations and study cellular heterogeneity [36]. CyTOF can assess not only surface markers but also intracellular proteins, enabling the study of cellular signalling pathways [37], epigenetic states [38] and the functional states of leukaemic cells and their immune microenvironments. In the last decade, CyTOF has been widely used for the immunophenotyping of haematopoietic stem cells after bone marrow transplantation (HSCT), as well as the immune monitoring of patients receiving cancer immunotherapy [39]. In the field of leukaemia, CyTOF has been pivotal in capturing the intratumour heterogeneity, identifying AML stem-like cells with unique signalling profiles [40] and subpopulations of B-ALL cells with activated pre-BCR signalling that are more resistant to standard therapy [41] and predictive of relapse [42,43]. More recently, CyTOF has been also used to characterise the metabolic regulome of single cells (scMEP) together with their phenotypic identity [44], revealing the metabolic dependencies of both leukaemic cells [45] and effector T-cells within the tumour microenvironment [46,47]. To enhance the study of the tumour architecture and immune system activity within the bone marrow niche, CyTOF can be combined with a high-resolution imaging system (IMC, imaging mass cytometry) able to resolve the spatial distribution of multiple proteins within the tumour and its microenvironment, enabling discoveries in terms of cellular interactions and disease progression [48]. The high-dimensional nature of mass cytometry has tangibly pushed the development of methods to analyse multiparametric data (either mass- or flow-based) at a single-cell level by performing dimension reduction [49], such as viSNE [50], UMAP or optSNE [51], or clustering methods, such as FlowSOM [52], PhenoGraph [42] or X-shift [53]. Although this technology has revolutionised the number of proteins that can be measured simultaneously, allowing the reliable detection of about 50 markers at a single-cell level, the selection of the markers and the optimisation of the panel can be trivial, specifically for the discovery of new druggable targets. Challenges in marker selection include ensuring antibody specificity, optimising the staining protocols and minimising signal spillover. Careful panel design is essential to account for the detection sensitivity across different mass channels. Key factors to consider include the sensitivity range of the detection instrument for isotopes, the intensity of surface marker expression, variations and expression patterns across samples and background noise or signal spillover [54]. CyTOF operates most sensitively within the atomic mass range of 153–176, making isotopes in this range ideal for antibodies targeting weakly expressed markers. Antigens are classified into primary (high expression), secondary (medium/variable) and tertiary (low/unknown) categories, similar to fluorophore-based flow cytometry panel design [54]. Antibodies for tertiary antigens or with low binding affinity should be paired with isotopes in the high-sensitivity range, while primary and secondary antigens can use isotopes outside this range. Mass cytometry reduces the background compared to conventional flow cytometry, but signal spillover remains a challenge. Spillover arises from isotope abundance sensitivity, where high-expression antigens broaden the mass peaks, and oxide formation arises from certain isotopes (e.g., La, Nd, Sm), generating signals at M + 16. Machine calibration minimises oxidation, and spillover matrices assist in addressing these issues. Impurities in isotopes also contribute to signal overspill, but these can be mitigated during setup [55,56].

In contrast, other proteomics platforms, like mass spectrometry, provide unbiased, high-throughput protein identification and quantification, but single-cell resolution is still challenging. Each platform has unique strengths and limitations regarding marker selection and reproducibility. Integrating data from multiple platforms can offer comprehensive insights into complex biological systems.

#### 2.3.2. Single-Cell Proteomics by Mass Spectrometry (MS)

Proteomics studies have been always performed in bulk systems, with the risk of missing low-abundance heterogeneous proteins. The first single-cell proteomics (SCP) attempts were limited to cells with richer proteomes (e.g., Xenopus egg) [57]. In SCP, the greatest impediment is the lack of a PCR equivalent in amplifying low-abundance proteins and the inability to adopt a “barcode” strategy to identify sequenced proteins [58]. The current technical developments are reported to possess enough sensitivity to resolve cells with minute differences and proteomics profiles. This ability is highly needed in the diagnostic field, where proteomics analysis could help in identifying diagnostic and therapeutic biomarkers. Schoof et al. managed to analyse the heterogeneous differentiation hierarchy in the OCI-AML8227 AML cell line [59]. Using SCP, they captured 1000 proteins, which helped in identifying three cellular subpopulations: self-renewal LSCs (CD34+, CD38−) (leukaemic stem cells), non-self-renewing progenitors (CD34+ CD38+) and mature differentiated blasts (CD34−). They suggested that their workflow could be potentially translated and applied to patients’ samples to characterise tumoural subclones. At present, SPC is not widely applied across laboratories due to the limited accessibility to workflows, which are often “lab-made” and not commercially available. For instance, some workflows require customised chips (e.g., SciProChip [60], ProteoCHIP [61], OAD chip [62], nanoPOTS chip [63]). SCP is mainly performed on liquid tissue, such as liquid biopsies, while tissue disaggregation procedures for solid tumours could cause undesirable protein expression changes [64]. Still, SCP could represent a huge step forward in the development of a comprehensive picture of the cellular state, integrating all the different omics information.

### 2.4. Metabolomics

Lately, growing interest has been focused on how the metabolic repertoire of cells can influence their fate and development. Changes in metabolic features are no longer considered only bystander effects of genetic instability but active players in disease progression and the response to therapy. Mitochondrial oxidative phosphorylation has recently been identified as a biological property that influences the response to antitumour therapy in AML [65]. In other haematopoietic diseases, such as multiple myeloma, electron transport chain activity is also a predictor of venetoclax sensitivity [66]. Since mitochondrial energy metabolism often shifts between diagnosis and relapse, a reliable method to measure this variable is crucial to predict the drug response, especially in clinical trials. The freshly extracted blast can easily suffer from stress, a condition that should be evaluated before determining oxidative phosphorylation (OXPHOS). A standardised, fast and robust protocol to measure mitochondrial oxygen consumption in blasts would allow us to determine whether OXPHOS might be a predictive biomarker of the drug response, therefore supporting AML management in future clinical trials [67]. In the next section, we explore the use of extracellular flow analysers to measure blast oxygen consumption.

#### 2.4.1. Seahorse—Bulk Analysis

The Agilent Seahorse XF Analyzer is a real-time platform that allows the analysis of cellular energy metabolism. This instrument offers a comprehensive overview of cellular respiration, reporting, from each assay well, the oxygen consumption rate (OCR), proton efflux rate (PER) or extracellular acidification rate (ECAR) and ATP production rates. The two primary methods for cellular energy production are mitochondrial respiration and glycolysis, which involve the consumption of oxygen and the release of protons, respectively. The Seahorse XF technology employs label-free sensors to monitor extracellular changes in these substances, thereby measuring the cellular respiration rates, glycolysis and ATP production [68]. Mitochondria respiration can switch from basal to maximal respiration performance, with the difference between the two defined as the spare respiratory capacity (SRC). The latter is considered an indicator of mitochondrial plasticity as the capacity of mitochondria to respond to pathophysiological conditions. The SRC levels vary across blood cells and are linked to different conditions, with lower levels in rapidly proliferating tumour cells and higher levels in differentiated cells with high ATP needs, like cardiomyocytes and hepatocytes [69]. AML cells exhibit lower SRC levels compared to peripheral mononuclear cells, despite having a greater mitochondrial mass [70]. However, SRC data are often obtained from stable leukaemic cell lines cultured in vitro. The data should be validated in primary blasts, although challenges such as limited patient sample availability and issues with experimental reproducibility may arise. Additionally, patients’ characterisation by Seahorse could provide critical translational insights, enabling a deeper understanding of metabolic vulnerabilities that may guide targeted therapeutic strategies.

When conducting Seahorse analysis, various energetic factors must be considered. This methodology shows limitations, such as the need for standardisation, quality control and normalisation. Bioenergetic experiments can vary due to factors like the growth conditions, cell viability and origin. Although methods like protein concentration or cell counting are suggested for normalisation, no single optimal factor exists [71]. In conclusion, the Seahorse technology represents a fast and easy method to obtain a preliminary screenshot of the metabolic cellular state. However, this instrumentation does not allow discrimination at a single-cell level or the detection of intratumour heterogeneity.

#### 2.4.2. Single Cell Energetic Metabolism by Profiling Translation Inhibition (SCENITH)

In an attempt to translate the information from Seahorse at a single-cell level, Argüello et al. developed Single Cell Energetic Metabolism by Profiling Translation Inhibition (SCENITH), a functional assay to quantify the metabolic dependencies and capacities of several cell types with a single-cell resolution, overcoming the challenge of performing cell sorting [72]. Specifically, Argüello et al. identified the protein synthesis (PS) machinery as one of the most energy-consuming processes in cells, representing a methodological opportunity to determine the protein synthesis levels as a measure of the global metabolic activity [72]. They took advantage of puromycin, combined with a novel anti-puro monoclonal antibody detectable by flow cytometry. Puromycin is an antibiotic protein synthesis inhibitor that causes premature chain termination during translation and, for this reason, is a reliable readout for the measurement of protein synthesis levels [73,74]. The core principle of SCENITH is that protein synthesis, as measured by puromycin incorporation, is closely linked to ATP production. This correlation was confirmed by observing a reduction in PS following the inhibition of major ATP sources using the ATP synthase inhibitor oligomycin and the glucose analogue 2DG [72]. Although this method is very promising, it has encountered some limitations, including the challenge of studying cells with very low protein synthesis levels [72]. Furthermore, analogously to Seahorse, this analysis is performed at a steady state, excluding the possibility of measuring the dynamic flux changes. Finally, the combination of SCENITH with sequencing technologies is still challenging, due to the fixation and permeabilisation steps required for anti-puro staining. Despite these limitations, SCENITH gives the opportunity to study energy metabolism at the single-cell level using standard instrumentation such as FACS, and it may potentially be combined with a panel of phenotypic markers. Moreover, unlike Seahorse, the SCENITH technology enables the detection of metabolic heterogeneities within a specific sample. Argüello et al. propose that SCENITH’s results could be helpful in analysing how factors such as diet, age and the anatomical or tissue context influence the energy metabolism of specific cellular subsets. Furthermore, SCENITH data may aid in defining a signature for tumour fitness, providing valuable insights for the prediction of patient outcomes and stratification of individuals for personalised therapeutic approaches [72].

#### 2.4.3. Single-Cell Metabolomics

Single-cell mass spectrometry allows the identification of specific clusters of metabolites or proteins with the possibility of a deep level of resolution, offering insights into clonal subpopulations potentially linked to intratumoural chemotherapy resistance. This makes metabolic and proteomic approaches valuable tools for the study of clonal heterogeneity. Metabolomic profiling, by measuring low-molecular-weight metabolites, can give a panoramic view of the pathophysiological cellular state. Single-cell metabolomics further enhances the analysis of cellular heterogeneity at the individual cell level, distinguishing unique subpopulations. Metabolomic studies are usually performed with MS or nuclear magnetic resonance (NMR) techniques [75]. Structural complexity, a high resolution and the sensitivity of cellular systems are essential features to consider when performing metabolomic studies. Single-cell metabolomics requires precise single-cell isolation methods and highly sensitive instruments [75], since the scMS workflow does not include a signal amplification step due to the great structural metabolic diversity.

Single-cell MS can be categorised into two groups: imaging MS and live single-cell MS (LSC-MS) [75]. The most common imaging MS methods are matrix-assisted laser desorption/ionisation mass spectrometry (MALDI-MS) and laser ablation electrospray ionisation mass spectrometry (LAESI-MS). Required sample processing with a vacuum for MALDI-MS or with an infrared laser beam for LAESI-MS could generate a potential metabolic perturbation before the analysis. The main goals that these technologies were developed for were minimising perturbation and ensuring quantitative performance.

The sample isolation step represents the main limitation of LSC-MS, since obtaining a high number of cells to gain statistically significant data is challenging. scMS methods are often complicated and they focus on achieving high sensitivity, with less focus on robustness and reproducibility [76]. This issue is exacerbated by the lack of a standardised protocol in reporting and integrating biological data. Moreover, the single-cell metabolome does not have a database to refer to, and high biological variability is intrinsic in the experiment. Raw data could be processed by “Pathway Activity Profiling” (PAPi) to predict dysregulated metabolic pathways [77]. Data obtained from the different omics technologies could be integrated in a cross-omics attempt to capture the full biological picture. Moreover, it has been proposed that metabolomics can improve biopsies’ classification in several tumoural malignancies [78], and these data could be applied for more accurate patient stratification.

#### 2.4.4. Single-Cell Metabolic State

The current single-cell proteomic techniques show limitations due to their relatively low throughput, requirements for custom mass spectrometry instrumentation, challenges in the analysis of cells cultured at a high density and the lack of computational methods for downstream analysis. Altogether, these constraints have led to the limited accessibility of single-cell metabolomics. A key challenge of MALDI-based single-cell metabolomics is the assignment of metabolite intensities to individual cells. Therefore, there is an urgent need to simplify and make both the techniques and the data currently available more accessible. Recently, Rappez et al. developed SpaceM, a method that does not require a custom mass spectrometry setup but rather uses commercially available MALDI imaging mass spectrometry and light microscopy [79]. SpaceM integrates MALDI imaging with light microscopy, followed by image segmentation and registration. Ultimately, SpaceM generates a spatio-molecular matrix for each cell, containing a normalised metabolic profile and microscopy-derived phenotypic properties such as cell fluorescence intensities and morpho-spatial features. SpaceM detects > 100 metabolites and lipids from >1000 cells per hour, demonstrating high sensitivity and throughput. The single-cell resolution of SpaceM was validated on a model of co-cultured human epithelial cells (HeLa) and mouse fibroblast cells (NIH3T3), where the cell types were predicted with accuracy of 91.3% solely based on the single-cell metabolomes. Although this method shows great potential, to date, the SpaceM method has been exploited only for the single-cell metabolomics of cultured cells. We envision the further applicability of this method to characterise the metabolic architecture of the leukaemic bone marrow niche, where different cell types coexist and interact.

A summary of the information regarding the described technologies is shown in Table 1.

The methodologies described so far have been developed to detail the architectures of complex and heterogeneous tumours such as leukaemia. Focusing on technologies with higher definition will allow researchers to describe the complete spectrum of leukaemia’s features at a single-cell level (genetic, epigenetic, metabolic). Several metabolic dependencies of leukaemia have been already identified, and the increased definition of these techniques will help to define new targetable metabolic routes. In the next section, we will describe the main metabolic drivers of the energetic cellular processes that are often dysregulated in leukaemia.

## 3. From Advanced Technologies to the Exploration and Identification of Shared Metabolic Pathways

Acute leukaemias are a notoriously heterogeneous group of haematopoietic neoplasms, and this variety has important implications in disease diagnosis, patient stratification and the therapeutic approach. As the biology of AML and ALL is further characterised, a wide array of deregulated pathways that drive the proliferation of blasts are being discovered. As already introduced in the previous paragraph, rewired cellular metabolism has become one of the most important hallmarks of cancer.

### 3.1. Glycolysis

Leukaemia, as with many other cancer cells, is in constant need of nutrients to fuel anabolic processes and to sustain its increased proliferation rate. Glucose is the primary energy source and precursor of several building blocks in mammalian cells [80]. Aerobic glycolysis becomes, therefore, the preferential means by which cells generate energy in the form of ATP, NADH and pyruvate, which is preferentially reduced to lactate (Warburg effect). Mitochondrial respiration is perfectly functional, but the preference towards aerobic glycolysis is sustained by the increased expression of the genes of glucose transporters and glycolytic enzymes, aberrantly regulated by oncogenes [81,82]. The intermediates and products of glycolysis can have complex roles. They can contribute to anabolic processes such as de novo nucleotide, amino acid and lipid synthesis [83,84,85], but also to the remodelling of the microenvironment and the promotion of metastasis [86]. Lactate, as the product of glycolysis, can have cell-intrinsic functions, influencing the expression of lactate transporters and lactate dehydrogenase B (LDHB) with the function of converting it to pyruvate for the tricarboxylic acid (TCA) cycle. Moreover, lactate has dynamic roles in the surrounding microenvironment as it can inhibit cytotoxic T-cells (L-lactate) and polarise macrophages towards M2. On the other hand, D-lactate can enhance T-cell function by stimulating the electron transport chain (ETC) [87]. Accelerated glycolysis due to the upregulation of transporters (GLUT 1–5) and enzymes is a hallmark of chemotherapy resistance in AML [88]. Additionally, the upregulation of glycolysis mediates prednisolone resistance in ALL [89].

### 3.2. Mitochondrial Metabolism

Mitochondria are the main organelles devoted to energy production and the synthesis of macromolecular precursors in the cell.

The energy demand of cancer cells is satisfied through the production of ATP derived from different metabolic pathways, which firstly converge to the TCA cycle and finally OXPHOS, powered by the ETC located in the mitochondria. The TCA cycle provides the cofactors NADH/H^+^ and FADH_2_ to fuel OXPHOS [82] and requires a constant supply of acetyl-CoA, which can be synthesised from different sources (e.g., carbohydrates, lipids, proteins). In the case of an imbalance in the production of acetyl-CoA, the TCA cycle can be fuelled by anaplerotic reactions [90]. Anaplerosis is essential for cells, especially for leukaemic ones, to sustain cellular growth in case of energy insufficiency. It has been recently demonstrated that oxidative and reductive reactions (such as proline and ornithine synthesis) occur within the same organelle in response to changes in nutrient availability and the bioenergetic demand, coordinating OXPHOS, amino acid synthesis and one-carbon metabolism [91].

Multiple mechanisms of mitochondrial dysregulation have been reported to cause the proliferation of leukaemia clones, including higher mitochondrial DNA (mtDNA) content, lower mitophagy, the evasion of apoptosis and metabolic shifts from the Krebs cycle (TCA) to fatty acid oxidation (FAO).

#### 3.2.1. Regulation of Mitochondrial Dynamics in Acute Leukaemias

Mitochondrial pathways contribute to the survival of LSCs; therefore, several aspects of mitochondrial energy metabolism could be potential targets for therapeutic intervention. AML LSCs display a unique mitochondrial morphology due to the expression of mitochondrial fission protein 1 (FIS1) [92]. Importantly, FIS1 overexpression is associated with a poor prognosis in AML patients and could be considered as an indirect marker of the LSC signature [92]. The inhibition of pyruvate mitochondrial carrier (MTCH2) has been linked to AML LSCs’ survival and differentiation [93]. The blockage of MTCH2 leads to an increase in pyruvate in the cytoplasm and the higher activation of pyruvate dehydrogenase in the nucleus, promoting histone acetylation and LSC differentiation and reduced survival [93]. In 2015, Saito et al. demonstrated that haematopoietic stem cells (HSC) reduced LSCs’ function through reducing the glycolysis dependency and increasing the reactive oxygen species (ROS) in AML mouse models [94]. Moreover, they demonstrated that the loss of 5′ AMP-activated protein kinase (AMPK) function reduced OXPHOS [94], and, upon dietary restriction, AMPK was activated to maintain the leukaemic potential. The latter work clearly demonstrates that LSCs can regulate metabolic pathways depending on the condition in which the LSCs exist. Furthermore, LSCs use mitochondrial protective mechanisms towards oxidative stress, such as the overexpression of Bcl-2 family proteins, to prevent the release of apoptotic factors, making them resistant to standard treatments [95]. In mature AML blasts, glucose deprivation decreases the viability in culture, indicating that the energy pathways in mature blasts and in LSCs are different, supporting metabolic heterogeneity within cancer. Mitochondrial metabolism is widely explored as a target for anticancer treatment in T-ALL. Emerging strategies targeting mitochondria in T-ALL involve BH3 mimetics, a class of drugs that antagonise the anti-apoptotic Bcl-2 family proteins, with venetoclax as first in line among them. Voltage-dependent anion channel (VDAC)1-directed drugs are also considered, which promote the suppression of aerobic glycolysis, VDAC1 closure, mitochondrial Ca^2+^ overload, the cessation of oxidative phosphorylation, oxidative stress and the release of proapoptotic factors [96].

#### 3.2.2. “ROS Low” vs. “ROS High”: A Delicate Balance

The balance between glycolytic and oxidative metabolism in haematopoietic cells is delicate, with cells shifting between metabolic states depending on their functional status. HSCs, for example, are primarily glycolytic and display low OXPHOS activity when in a dormant state, albeit carrying a relatively high number of mitochondria [97,98,99,100]. Within the hypoxic bone marrow (BM) niche, HSCs rely on anaerobic glycolysis, which minimises ROS production and protects cells from oxidative damage. Additionally, HSCs with low ROS levels show enhanced primary and secondary engraftment potential compared to their high-ROS counterparts. In contrast, the LSCs in AML are heavily reliant on mitochondrial metabolism for survival, exhibiting elevated OXPHOS activity and distinct gene expression profiles compared to non-malignant HSCs [101,102,103,104]. Functionally defined LSCs in AML are characterised by low levels of reactive oxygen species (ROS low) and the aberrant expression of BCL2 apoptosis regulator (Bcl-2), which is responsible for the maintenance of OXPHOS [105]. Furthermore, they are uniquely dependent on amino acids for survival, distinguishing them from ROS-high blasts and healthy HSCs.

A low O_2_ concentration promotes a shift from mitochondrial OXPHOS to glycolysis to reduce oxidative stress. Studies on AML cell lines and primary blasts confirm that hypoxia predominantly alters energy production pathways, but the results depend on cell-specific factors [106]. For instance, while some cell lines exhibit similar glycolytic adaptations under hypoxia, others show distinct metabolic changes influenced by their genomic background. In AML models, hypoxia-driven metabolic reprogramming includes increased glycolysis through the upregulation of glycolytic enzymes, as well as alterations in TCA cycle metabolites, modifying the mitochondrial components to mitigate ROS-induced damage. These adaptive mechanisms are influenced by the genomic and cellular contexts. The metabolomic profiling of primary AML cells revealed a hypoxia-associated signature, including elevated levels of lactate, pyruvate and 2-HG, which correlated with poorer clinical outcomes [107]. This underscores the critical role of metabolic shifts in leukaemic stem cell survival in hypoxic BM niches and highlights the need for further research into how the genetic background drives these adaptations.

Drug-resistant clones demonstrate metabolic reprogramming [20], which contributes to resistance against therapies targeting Bcl-2 and induced myeloid leukaemia cell differentiation protein (MCL1) [108]. Identifying these specific metabolic vulnerabilities could pave the way for targeted therapeutic strategies to eradicate the LSC compartment in acute leukaemia. A high steady state of mitochondrial calcium import was recently identified as a vulnerability in venetoclax-resistant AML [109]. Inhibition of the calcium importer (MCU) was shown to eradicate the resistant LSCs by sharply reducing OXPHOS, making this an interesting target for novel therapies.

### 3.3. Amino Acid Metabolism

**Figure 1:**
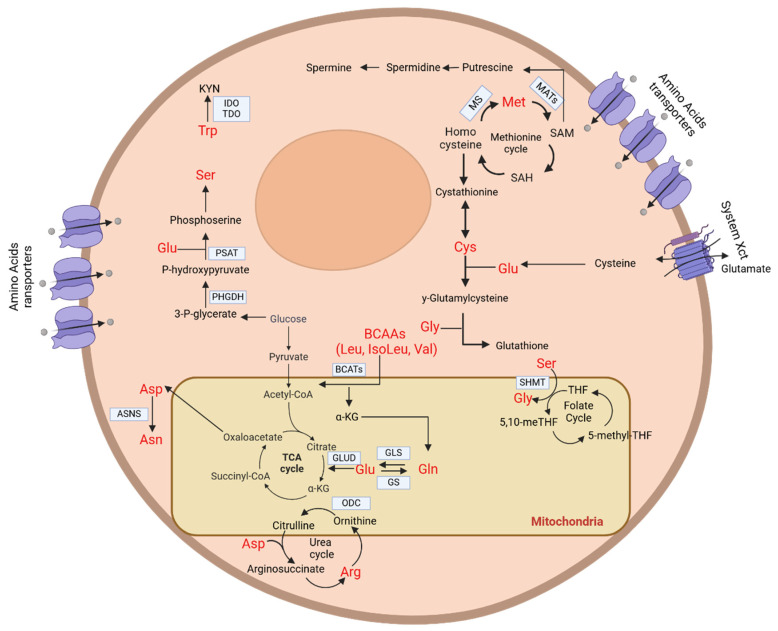
Amino acid pathways dysregulated in acute leukaemia and potentially addressable with targeted molecules. 5,10-Methylenetetrahydrofolate (5,10-mTHF); Alpha-Ketoglutarate (α-KG); Arginine (Arg); Asparagine (Asn); Asparagine-Synthase (ASNS); Aspartic Acid (Asp); Branched-Chain Amino Acids (BCAAS), Mitochondrial BCAA-Transaminases (BCATs); Cysteine (Cys); Glutamine (Gln); Glutaminase (GLS); Glutamate (Glu); Glutamate Dehydrogenase (GLUD); Glycine (Gly); Glutathione Synthase (GS); Indoleamine 2,3 Dioxygenase (IDO); Leucine (Leu); Isoleucine (IsoLeu); Kynurenine (KYN); Methionine-Adenosyl-Transferase (MAT); Methionine (Met); Methionine Synthase (MS); Ornithine-Decarboxylase (ODC); Phosphoglycerate Dehydrogenase (PHGDH); Phosphoserine Aminotransferase (PSAT); S-Adenosyl-Homocysteine (SAH); S-Adenosyl-Methionine (SAM); Serine Hydroxymethyltransferase (SHMT); Tricarboxylic Acid (TCA); Tryptophan-2,3-Dioxygenase (TDO); Tetrahydrofolate (THF); Tryptophan (Trp); Valine (Val). Created in BioRender. Tettamanti, S. (2025) https://BioRender.com/d64v911 (accessed on 18 December 2024).

Amino acid metabolism has been acquiring increasing importance in the acute leukaemia translational research landscape in recent years. Amino acids (AA) are critical players in the cellular metabolic setting, not just for their central role in protein biosynthesis regulation but also for their secondary functions as redox balance regulators, as well as being alternative fuels for energy production and derivative production (Figure 1). These derivatives are involved in a plethora of functions and signalling pathways that ultimately regulate cellular homeostasis. Malignant cells often demonstrate important changes in their metabolism, making this a potential therapeutic target. The main point associated with targeting cellular metabolism is the differentiation between healthy and malignant cells [110]. Specifically, the ability to synthesise non-essential amino acids (NEAAs) is lost during cancer progression, while maintained by healthy cells. This ensures that non-malignant cells are unaffected by potential enzymatic depletion or restrictions of amino acids [110]. The many different therapeutic strategies that are currently being developed for the treatment of acute leukaemia include targeting the metabolism of essential, conditionally essential and non-essential AA [111,112].

#### 3.3.1. Essential Amino Acid Metabolism

##### Methionine

Aside from being the precursor of the universal methyl donor S-adenosyl-methionine (SAM), methionine (Met) is also heavily involved in one-carbon (1C) metabolism and in polyamine biosynthesis. Met can be imported by different transporters (SLC6A14, SLC6A19, SLC38A1, SLC38A2, SLC7A5, SLC7A7 and SLC1A5) with varying affinities; thus, the inhibition of Met import is considered an impractical therapeutic approach. Additionally, tumoural proliferation and expansion appear to be dependent on exogenous Met, and this dependency has been defined as the Hoffman effect [113]. In the last century, Hoffman et al. demonstrated that malignant cells were able to synthesise high levels of Met endogenously, yet they were insufficient to maintain cancer growth [113]. The first experiments on Met metabolism were conducted in the previous century. Bone marrow cells from non-leukaemic patients needed a lower concentration of L-Met in the culture medium compared to bone marrow cells from leukaemic patients. The cytoplasmic pool size of L-Met in haematopoietic cells was approximately twice as high in normal subjects compared to leukaemia patients [114]. Moreover, bone marrow cells from leukaemia patients were found to be more sensitive to methioninase compared to those from patients with non-malignant conditions [115]. Mechanistically, intracellular Met is converted to SAM via an ATP-dependent reaction catalysed by the methionine-adenosyl-transferase (MATs) enzyme family, often upregulated in AML [116]. SAM, a universal methyl donor, leads to histone and DNA hypermethylation, and its demethylation produces S-adenosyl-homocysteine (SAH). SAH is hydrolysed into homocysteine, which can be recycled back into Met or enter the transculturation pathway. SAM is also involved in polyamine biosynthesis, generating spermidine and 5′-methylthioadenosine (MTA), which can further regenerate Met [111]. Spermidine is a metabolite specifically enriched in LSCs, and reducing the spermidine or total polyamine concentrations could impair LSC function by downregulating the expression of lysine acetyltransferase 7 (KAT7). Therefore, polyamine depletion could represent a potential therapeutic approach to prevent relapse for patients with AML [117].

##### BCAAs (Leucine/Isoleucine/Valine)

Branched-chain amino acids (BCAAs) play an important role in modulating the intracellular availability of α-KG through glutamate as an intermediate. BCAAs are imported through high-affinity transporters (SLC7A5, SLC7A8, SLC43A1 and SLC7A9), which are typically upregulated in AML. SLC7A5, SLC7A8 and SLC43A1 mediate the exchange of intracellular glutamine and extracellular leucine. In AML, a reduction in intracellular glutamine leads to the inhibition of mTORC1 complex targets, inducing apoptosis, whereas intracellular leucine mediates mTORC1 activation, promoting proliferation, cell growth and survival in AML, as well as in other malignancies [112,118]. Human AML LSCs have been reported to upregulate BCAA-transaminase 1 (BCAT1) expression [119]. BCAT1 overexpression has been shown to deplete the intracellular α-KG levels, thus inhibiting Tet methyl cytosine dioxygenase (TET) and α-KG-dependent dioxygenases and leading to a general hypermethylated phenotype, similarly to the IDH 1–2-mutated setting. In IDH-mutated AML, BCAT1 is inhibited by the oncometabolite d-2-hydroxyglutarate (D2HG), leading to compensatory glutamate catabolism [120].

##### Tryptophan

Tryptophan (Trp) is metabolised by heme-dependent tryptophan-2,3-dioxygenase (TDO) and indoleamie-2,3-dioxygenase (IDO) into a precursor of the metabolite kynurenine (KYN) [112]. IDO is frequently overexpressed and secreted by AML blasts [121], leading to the accumulation of KYN, which has been reported to mediate a phenotypic switch in immune cells towards an immunosuppressive phenotype [122,123]. In IDH-mutant AML, KYN catalytic enzymes are often downregulated, leading to the further accumulation of this metabolite, overall worsening patients’ prognosis [124]. Correlation studies have been conducted also on other haematological diseases, such as adult T-cell leukaemia/lymphoma, peripheral T-cell lymphoma and diffuse large B-cell lymphoma, showing that the serum KYN, alone or in combination with the IDO levels, is an important prognostic factor [125,126,127,128]

#### 3.3.2. Conditionally Essential Amino Acid Metabolism

##### Glutamine

Glutamine (Gln) is the most bioavailable amino acid, and cancer cells frequently use it as an alternative fuel source. Glutamine is also used as a precursor for other amino acids, as a source of C and N and as a precursor for gluconeogenesis. Glutamine also allows the uptake of leucine through glutamine–leucine antiport systems, favouring cell proliferation and cancer growth through the upregulation of the mTORC1 pathway [129]. More rarely, Gln can be taken up via system L and y+ transporters, which mediates the glutamine–leucine exchange (see section on BCAAs), and, more frequently, by the neutral amino acid transporter SLC1A5. After uptake, Gln is converted into glutamate by enzymes characterised by Q-amido-transferase (GATase) domains or by mitochondrial glutaminase 1 or 2 (GLSs), frequently overexpressed in AML [130]. Gln plays a vital role in energy production, being converted into glutamate through GLS. Glutamate is further processed into α-KG via glutamate dehydrogenase (GLUD1) or transaminases. α-KG enters the TCA cycle or contributes to glutathione (GSH) synthesis, essential for cancer cells to counteract their elevated ROS levels [131]. In AML, the use of glutaminase inhibitor CB-839 has been linked to an increase in mitochondrial ROS (mitoROS), a reduction in GSH and cell death [132]. Additionally, GLS inhibition can be exploited as a target for AML and B-ALL by associating GLS inhibitors and mitoROS-inducing drugs. Furthermore, the IDH-mutated AML subtype is extremely sensitive to CB-839’s action [130]. Glutaminolysis is essential in NOTCH1-driven T-ALL cells, where Gln is converted to Glu and enters the TCA cycle, contributing significantly to its intermediates. Inhibiting WEE1, a kinase crucial for cell cycle checkpoints, renders cells dependent on glutaminolysis. Consequently, the dual targeting of WEE1 and GLS results in synergistic lethality in T-ALL [133]. Similarly, in AML cells, glutaminolysis may represent a therapeutic vulnerability when paired with specific tyrosine kinase inhibitors in FLT3-ITD-driven leukaemia [134]. Supporting this, a 2020 in vivo study by Van Gastel et al. investigated leukaemia’s dynamics during chemotherapy. By pinpointing the peak response time, isolating surviving cells and performing an unbiased metabolomics analysis, they revealed a unique metabolic adaptation in these cells. The surviving AML cells shifted towards glutamine utilisation to fuel pyrimidine and glutathione synthesis, rather than engaging the mitochondrial TCA cycle [135].

##### Cysteine

Cysteine metabolism is strictly correlated with the intracellular glutamate levels, since its import is mediated by the cystine (the oxidised form of cysteine)–glutamate exchanger (xCT). Cysteine metabolism has a central role in protein biogenesis and in the maintenance of cellular redox homeostasis, since it is necessary for the biosynthesis of GSH. Low intracellular cysteine levels lead to high ROS accumulation and metabolic shutdown, especially in highly metabolically active tumour cells. This makes cysteine an important metabolic source for cancer cells, especially for LSCs [136]. Interestingly, given its role in regulating intracellular ROS levels, it has been reported that the inhibition of xCT might lead to ferroptosis in AML cells [137]. Indeed, in 2022, Pardieu et al. demonstrated that inhibiting the xCT system with sulfasalazine enhanced the efficacy of frontline therapies, such as anthracycline daunorubicin, in AML, independently of the genetic background [138].

##### Serine and Glycine

Serine and glycine are involved in cellular redox homeostasis through GSH biosynthesis. These two amino acids are synthesised via the serine synthesis pathway (SSP), which is frequently upregulated in AML due to the increased expression of phosphoglycerate dehydrogenase (PHGDH) and phosphoserine aminotransferase 1 (PSAT1). In LSCs, it has been reported that PHGDH is upregulated through a CREB-dependent mechanism [139]. SSP is also involved in the production of TCA anaplerotic α-KG by consuming intracellular glutamate. Therefore, the inhibition of this pathway results in hindering cell growth and proliferation. In AML, SSP can be a metabolic vulnerability in specific nutrient conditions. Fructose abundance in the BM of AML patients can trigger the upregulation of SSP, increasing their dependency on it, therefore making PHGDH inhibition effective in eradicating leukaemia [140]. Additionally, serine produced through SSP can be converted into glycine by the serine hydroxy methyltransferase (SHMT) enzyme in a tetrahydrofolate-dependent reaction. Both serine and glycine play crucial roles in one-carbon metabolism. Glycine can be decarboxylated by the glycine cleavage complex (GCS), resulting in the biosynthesis of 5,10-methylenetetrahydrofolate (5,10-mTHF), which is essential in regenerating methionine from homocysteine. Furthermore, mTHF serves as a vital intermediate in nucleotide biosynthetic pathways, linking amino acid and nucleotide metabolism. While it has been shown that inhibiting SHMT can lead to growth inhibition in chronic myeloid leukaemia (CML), there is still insufficient evidence regarding its effects in AML [111,112]. In T-ALL, genes associated with SSP, particularly the phosphoserine phosphatase (PSPH) enzyme, were found to be upregulated. Inhibiting PSPH expression in T-ALL cell lines induced cytostatic effects in vitro and reduced leukaemia’s expansion in vivo. While the mechanisms behind this vulnerability are still unknown, the targeting of PSPH has shown promise as a potential novel therapeutic approach for a subset of T-ALL patients [141].

##### Arginine/Ornithine and Polyamine Biosynthesis

Arginine plays a central role in the polyamine biosynthesis pathway, since its hydrolysis by cytosolic or mitochondrial arginases generates ornithine and urea [112]. Ornithine is a precursor of putrescine, which is generated by ornithine-decarboxylase (ODC) through ornithine decarboxylation. Putrescine is the precursor of polyamines such as spermidine and spermine, which are cationic proteins that control cell functions such as growth, proliferation, differentiation, motility and survival via their interactions with negatively charged macromolecules such as nucleic acids or proteins. In AML, mitochondrial arginases are typically very active, leading to the high consumption of arginine from cancer cells and the consequent depletion of extracellular arginine. This can have repercussions regarding the homeostasis of T-cells and other immune cells in the tumour microenvironment [142,143]. Additionally, in 2015, Miraki-Moud et al. demonstrated that arginine deprivation induced by arginine deiminase, which catalyses the conversion of arginine into citrulline, significantly reduced the percentage of AML cells in mouse bone marrow [143]. Moreover, they combined arginine deiminase with cytarabine, a chemotherapeutic agent, and this approach increased cytarabine’s cytotoxic activity [143].

#### 3.3.3. Non-Essential Amino Acid Metabolism

##### Asparagine

Asparagine is synthesised through the transamination of aspartic acid by the cytosolic asparagine synthase (ASNS). Importantly, certain subsets of AML exhibit downregulated expression of the ASNS gene, making them more sensitive to asparaginase treatment, such as L-asparaginase [144]. Asparagine depletion by asparaginase induces cellular stress by activating the unfolded protein response, ultimately leading to cell death [112]. Furthermore, asparagine plays a critical role in regulating the antiport of other amino acids, such as glutamine, histidine, arginine and serine. In the bone marrow niche, asparagine is released from mesenchymal stromal cells and/or macrophages, which can have an important role in AML desensitisation towards asparagine depletion therapies [111,145]. In the example of ALL, one of the key features of lymphoblast deficiency is asparagine synthetase. Consequently, asparaginase (ASNase) has been developed for ALL treatment. Asparaginase catalyses the hydrolysis of serum asparagine into aspartate and ammonia, decreasing the levels of amino acids and inhibiting leukaemic cell growth [146]. In B- and T-ALL cell lines, sensitivity to ASNase was initially linked to the ASNS protein levels and not mRNA levels. However, a more complex picture emerged from a study involving both ALL cell lines and diagnostic samples. Unlike cell lines, ALL blasts express very low levels of ASNS, making the correlation between its expression and ASNase sensitivity uninformative [147]. Recent findings have shown that the methylation of CpG islands in the *ASNS* gene is an epigenetic mechanism leading to *ASNS* gene silencing in B-ALL. Higher *ASNS* methylation is associated with increased ASNase sensitivity due to reduced ASNS transcript and protein levels [148].

### 3.4. Lipid Metabolism

Among the most thoroughly investigated biomolecular families in cancer metabolism over the past decade are lipids. Lipid biosynthesis and catabolism are pivotal processes for cell growth and survival, favouring resilience to cellular stress, organelle and membrane synthesis and energy production. In cancerous settings, not only neoplastic blasts but also niche resident adipocytes frequently display alterations in lipid metabolic pathways. Adipocyte proliferation, growth and survival are frequently enhanced in AML, leading to increased lipolysis and establishing a metabolic crosstalk that supplies additional energy reserves to neoplastic cells. This highlights the significant influence of the interplay between cancer and these niches regarding lipid metabolism [111,149,150,151].

#### 3.4.1. Fatty Acid (FA) Metabolism

FA catabolism in AML is often aberrantly upregulated, since, in AML, FA are frequently consumed through the B-oxidation process to produce energy [111]. In AML, rate-limiting enzymes such as carnitine palmitoyl transferase 1a (CPT1a) and carnitine transporter 2 SLC22A16 (CT2) are often overexpressed and are validated therapeutic targets [4]. In terminally differentiated cells, the FA biosynthetic pathways are usually shut down to allow the consumption of FA derived from the diet to produce energy. In contrast, specific subtypes of AML, particularly IDH-mutated AML, can display aberrantly upregulated FA biosynthesis. This is achieved through the upregulation of key enzymes in FA anabolic processes, such as ATP-citrate-lyase (ACLY) and stearoyl CoA desaturase 1 (SCD1), although clinical evidence supporting a favourable effect of their inhibition is still lacking in AML treatment [152,153]. The upregulation of FA synthesis was correlated with therapy resistance in ALL. The increased expression of the FA synthase gene (*FASN*) was significantly greater in drug-resistant patients compared with therapy responders, suggesting that FASN inhibition can overcome a poor response to dexamethasone [154].

#### 3.4.2. Sphingolipid Metabolism

Sphingolipids, such as ceramides, sphingosine, dihydroceramide and sphingosine 1 phosphate (SP1), regulate different aspects of cellular homeostasis, such as signalling and signal transduction, cell growth, differentiation and death [111]. Strategies targeting sphingolipid metabolism have been recognised as potentially effective in AML treatment, especially given the evidence that specific AML oncogenes directly interfere with these metabolic pathways. Ceramides are bioactive sphingolipids generated de novo by ceramide synthetase (Ces 1–6), containing fatty acids of various lengths. The type of fatty acyl group that is attached often dictates the biological activity of ceramide. In AML, ceramides were shown to induce mitophagy-dependent cell death upon FLT3-ITD inhibition [155]. FLT3-ITD targeting resulted in the increased mitochondrial translocation of CerS1 and increased mitochondrial C_18_-ceramide generation. Mitochondrial C_18_-ceramide interacted with LC3B-II to recruit autophagosomes to mitochondria for degradation via the ceramide-binding domain. Additionally, AML can upregulate ceramide catabolism through acid ceramidase (AC) overexpression [155]. AC cleaves ceramides and allows for an increment in SP1 levels, which is also supported by an increment in SP1 biosynthesis by the sphingosine kinase enzyme, which is constitutively overexpressed in AML [156,157]. Ceramide regulation plays an important role in ALL as well, as the upregulation of ceramide synthase 6 (CERS6) was observed in T-ALL compared to peripheral blood mononuclear cells and normal T-lymphocytes. CERS6 is an isoform of ceramide synthase that is known to generate ceramides with C16 acyl chains (C_16_-Cer), and its overexpression mediates the resistance to ABT-737 in T-ALL [158]. To date, there are no direct inhibitors of CERS6, but its expression may serve as a biomarker in determining the effectiveness of anticancer agents acting via the extrinsic pathway in T-ALL.

#### 3.4.3. Mevalonate Pathway

Finally, sterols and isoprenoids represent another class of lipid molecules of interest for AML metabolic targeting. These molecules are synthesised through the mevalonate pathway, which uses acetyl-CoA to produce 3-hydroxy-3-methylglutaryl-coenzyme A (HMG-CoA). HMG-CoA is then converted into mevalonate by the rate-limiting enzyme HMG-CoA reductase. The inhibition of this rate-limiting enzyme was shown to induce apoptosis in malignant AML cells [159]. In vitro studies have pointed out that TAL1-positive T-ALL cell lines also upregulate the mevalonate pathway via MYCN gene expression, suggesting that HMGCR inhibition is a potential therapeutic strategy [160].

**Figure 2:**
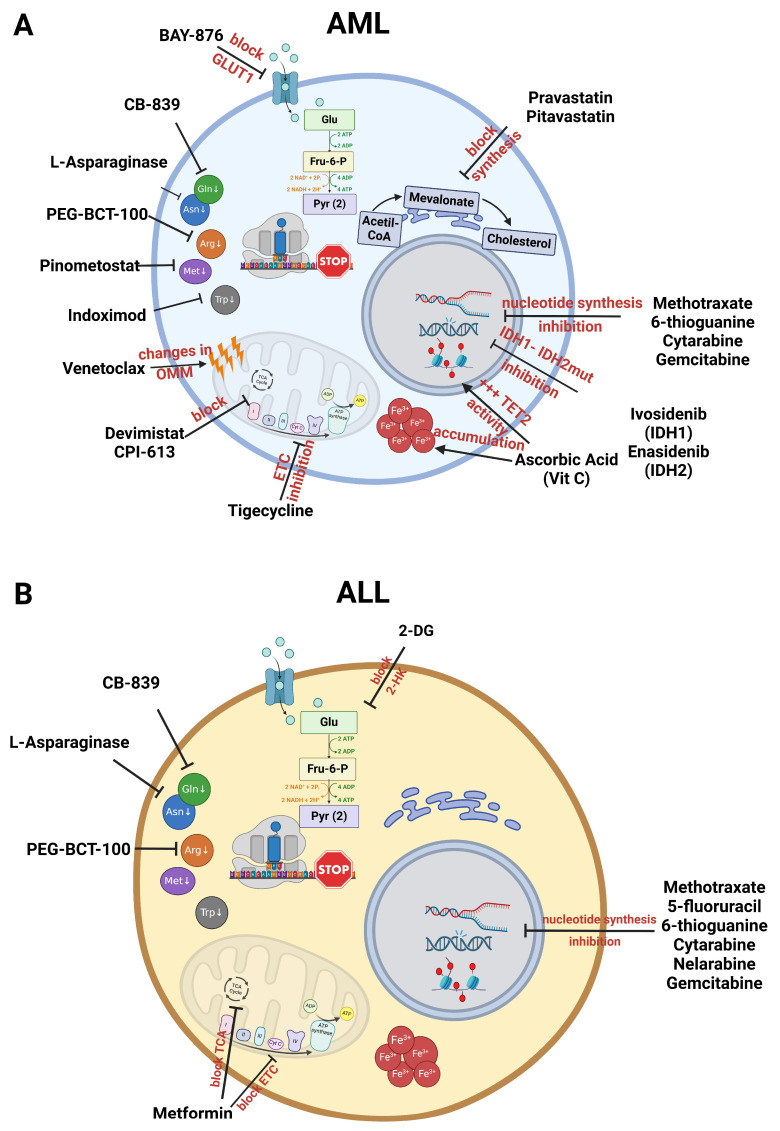
Action sites of clinically targeted molecules. Panel (**A**) shows targets in acute myeloid leukaemia (AML) and panel (**B**) in acute lymphoblastic leukaemia (ALL). 2-Deoxy glucose (2-DG); electron transport chain (ETC); glucose transporter 1 (GLUT1); isocitrate dehydrogenase (IDH); outer mitochondrial membrane (OMM); tricarboxylic acid cycle (TCA); Tet methyl cytosine dioxygenase 2 (TET2). Created in BioRender. Tettamanti, S. (2025) https://BioRender.com/l59b367 (accessed on 18 December 2024).

As previously discussed, methodologies and targetable metabolic pathways have been under the spotlight in translational medicine. In the final section, we will focus on clinical interventions designed to target these metabolic pathways in leukaemic cells. Figure 2 shows the action sites of the targeted molecules available and under clinical research.

#### 3.4.4. Metabolic Rewiring in Haematological Malignancies vs. Solid Tumours

Metabolic rewiring plays a pivotal role in cancer, although the specific patterns and impacts of this rewiring can differ between haematological malignancies and solid tumours. Blood cancers evolve in a dynamic, fluid context, where cancer cells circulate in the bloodstream or migrate to the BM or lymphatic tissue. As previously described, blood cancer cells, due to their dynamic and circulatory nature, upregulate pathways that support rapid proliferation and adaptation, such as glycolysis, amino acid metabolism and nucleotide biosynthesis. In contrast, solid tumour cells are embedded in dense, structured masses with a distinct microenvironment, where metabolic rewiring is driven by the tumour microenvironment (TME), which is shaped by the hypoxic conditions and nutrient gradients.

Despite these differences, there are notable similarities between the two contexts, since blasts cells grow in the BM niche, which also presents a structured TME, where the hypoxia and nutrient availability vary according to the disease stage. Through cellular interactions and cytokine signalling, the BM niche also influences the development and metabolic state of leukaemic stem cells.

Understanding these nuances within the TME is essential in developing targeted therapies for both blood cancers and solid tumours. The targeting of metabolic pathways must be tailored to the tumour’s nature and architecture. For blood cancers, new approaches focus on targeting glucose metabolism and glutamine and nucleotide synthesis. For example, the inhibition of glucose transporters like GLUT1 is being explored in AML [161]. Additionally, the targeting of metabolic genes such as IDH1–2 mutants has shown efficacy and is currently approved for both AML [162] and certain solid tumours [163]. In the case of solid tumours, therapeutic strategies targeting hypoxia-inducible factor 1-alpha (HIF1α) are also under investigation [164].

Ultimately, a deeper understanding of the metabolic landscape in different cancer types is critical for the development of more effective, targeted treatments.

## 4. Clinical Interventions (Clinical Trials): Drugs Acting on Metabolism in ALL and AML—How Far Are We?

Cancer has been historically treated with drugs that have metabolic implications. In the 1950s, antifolate agents [165] were the first drugs with antimetabolic effects that were successfully introduced to achieve remission in childhood ALL. Subsequently, several molecules with metabolic effects were introduced in the treatment of leukaemia, and many of these are still used in clinical practice nowadays (such as methotrexate, 6-mercaptopurine, etc.). These agents, summarised in Table 2, are well-known chemotherapy drugs with metabolic implications that are not specific to tumour cells and cause side effects, damaging healthy cells.

Therefore, more in-depth studies of metabolism regulation are leading to the identification of upregulated pathways in cancer cells, which may display metabolic vulnerabilities that could be further explored as successful targets of cancer therapy. Growing evidence reports the development of new therapies, tailored to target specific metabolic pathways, some of which have already been included in clinical trials, as summarised in Table 3.

### 4.1. Targeting Glycolysis

Targeting altered glycolysis through the inhibition of cell surface transporters or glycolytic enzymes is a promising strategy to target leukemogenesis. Preclinical studies in AML showed that the genetic ablation or pharmacological inhibition of GLUT1 with BAY-876 led to the suppression of leukaemia progression and improved the survival of AML transplanted mice. Moreover, the dual inhibition of GLUT1 and OXPHOS exhibited synergistic anti-leukaemic effects in AML patient samples via limiting their metabolic plasticity [161]. Another GLUT1 inhibitor studied in cancer treatment is STF-31, which has shown promising results in several solid tumour-derived cell lines [167]. Finally, glycolysis inhibition with the hexokinase 2 (HK2) inhibitor 2-deoxyglucose (2-DG), simultaneously with MCL1 inhibition, sensitised paediatric ALL cell lines towards prednisolone [89]. Although not yet clinical, the evidence reported suggests that glycolysis inhibition may be a means to target leukaemic cells.

### 4.2. Mitochondria

#### 4.2.1. Targeting ETC

Tigecycline is a glycylcycline that is used to treat complicated microbial infections. In haematological malignancies, tigecycline induces cell cycle arrest, apoptosis, autophagy and oxidative stress; its action is mediated by ETC complex inhibition, which blocks mitochondrial OXPHOS and cell proliferation [168]. The combination of tigecycline with chemotherapeutic or targeted drugs has been shown to be promising by inhibiting LSCs [169], and a phase I dose escalation study of tigecycline monotherapy in patients with R/R or newly diagnosed AML not eligible for induction chemotherapy has been successfully completed [170].

IACS-010759, an inhibitor of the ETC complex, disrupts OXPHOS. Moreover, in NOTCH1-mutated T-ALL cells, it inhibits growth via metabolic shutdown and redox imbalance, determining metabolic reprogramming into glutaminolysis [171]. Phase I trials in patients with R/R AML (NCT02882321) have been discontinued due to lactic acidosis and neurotoxicity; IACS-010759 showed a narrow therapeutic index, and further studies are needed to fully elucidate the toxicity mechanisms [172].

#### 4.2.2. Targeting TCA

CPI-613 (Devimistat) is a small-molecule inhibitor of mitochondrial metabolism, which inhibits pyruvate dehydrogenase (PDH) and prevents the entry of acetyl-CoA into the TCA cycle. A phase 2 clinical trial assessing Devimistat with Mitoxantrone and cytarabine suggested an association between the age-related decline in mitochondrial quality and autophagy and Devimistat sensitivity in R/R AML; however, these results need to be further confirmed [173].

#### 4.2.3. Apoptotic Agents

Venetoclax is a BCL2 inhibitor that induces the release of CytC and apoptosis through structural alterations of the mitochondrial membrane, leading to metabolic reprogramming that seems to be BCL2-independent [174]; moreover, in combination with the hypomethylating compound azacitidine, venetoclax targets LSCs’ metabolic vulnerabilities by inhibiting amino acid metabolism and OXPHOS [175]. This combination has shown promising clinical results. A phase 1b clinical trial in AML revealed the successful eradication of LSCs [176]; furthermore, increased survival was reported with the addition of venetoclax to azacitidine in AML patients ineligible for conventional chemotherapy [177]. Finally, venetoclax is also recognised as a valuable therapeutic option in the R/R setting of AML [178].

#### 4.2.4. Biguanides

Metformin is the most prescribed antidiabetic treatment worldwide. Retrospective population-based studies revealed that metformin was associated with a reduced risk of cancer and decreased cancer-related mortality [179,180]. A strong dependence on OXPHOS or mitochondrial dysfunction was associated with susceptibility to metformin in slow-growing cancer cells [181,182]. Moreover, biguanides enhanced anticancer immunity by enhancing CD8+ T-cell tumour infiltration in murine models [183] and T-cell-dependent antitumour activity [184]. Metformin administration was shown to blunt the tumour microenvironment’s immunosuppressive action by decreasing Treg infiltration both in vivo and in vitro [185,186] and by reducing the number and activity of myeloid derived suppressor cells (MDSCs) [187]. Most of the anticancer effect of metformin is related to solid tumours. However, clinical trials have been undertaken with the addition of metformin to chemotherapy in haematological malignancies such as R/R AML and ALL (Table 3).

### 4.3. Amino Acid Metabolism

L-asparaginase is a well-known antitumour metabolic therapy, employed in its pegylated form in the first-line treatment of childhood ALL since the early 2000s; by catalysing the deamination of L-asparagine, the enzyme depletes the asparagine and glutamine levels in lymphoblastic leukaemic cells, thus promoting their apoptotic death [188]. A phase 1 clinical trial is ongoing on myeloid leukaemia as well (NCT02283190).

BCT-100 is a pegylated recombinant human arginase that leads to the rapid depletion of arginine; its synergic action was described in vitro with dexamethasone in ALL [189]. Subsequently, successful results were observed in adult patients with AML, with an increase in the overall response in the arm receiving BCT100 in combination with cytarabine, compared to that receiving cytarabine only; moreover, the addition of BCT100 was well tolerated [190]. Finally, a phase 1/2 trial documented the clinical safety of arginine depletion with BCT100 in children with R/R ALL and AML [191].

Pinometostat targets methionine metabolism and has shown encouraging results in phase 1/2 trials on R/R AML or newly diagnosed patients with an 11q23 rearrangement. Two other molecules acting on AA metabolism have completed phase 1 clinical trials in AML: indoximod, which targets tryptophan, and CB-839, which targets glutamine.

### 4.4. Fatty Acid Metabolism

Statins display several anticancer effects: they inhibit proliferation, angiogenesis, metastasis and cancer stemness, while inducing oxidative stress, cell cycle arrest, autophagy and apoptosis [192]. Pravastatin and pitavastatin in combination with chemotherapy have shown favourable results in phase 1 and 2 trials on AML (Table 3).

### 4.5. Nucleotide Metabolism

Cancer cell growth and proliferation depend on de novo nucleotide synthesis from intermediates in the TCA cycle, glucose-derived ribose sugars from the pentose phosphate pathway (PPP) and amino acids that generate purines and pyrimidine nucleotides. The first drug inhibiting nucleotide metabolism was mercaptopurine, approved in 1953 for ALL and currently used in ALL treatment. Several molecules that inhibit nucleotide metabolism were subsequently approved by the FDA: methotrexate, 5-fluorouracil, 6-thioguanine, Ara-C, nelarabine and gemcitabine. Despite their effects in targeting cancer cells, being non-specific, these drugs also affect healthy cells, causing side effects. FF-10501 is a novel inhibitor of guanine synthesis that acts on inosine 5′-monophosphate dehydrogenase, which is overexpressed in acute myeloid leukaemia cells. While promising results were observed in terms of clinical activity and target inhibition in heavily pretreated patients with AML, phase 2a was discontinued due to increased mucositis events (NCT02193958).

### 4.6. Antioxidants

Vitamin C (ascorbic acid) is an antioxidant that interferes with the redox balance. As a cofactor of Fe^2+^ and αKG-dependent dioxygenases, it promotes TET2 activity to limit haematopoietic stem and progenitor cells’ (HSPCs) self-renewal and leukemogenesis. Ottone et al. showed low plasma levels of vitamin C in AML at onset/diagnosis, along with decreased intracellular cytoplasmic accumulation, suggesting its consumption by AML cells [193]; furthermore, intravenous vitamin C administration improved the blood cell counts and quality of life of relapsed AML patients [194]. Moreover, clinical remission following ascorbate treatment was described in a patient with TET2- and WT1-mutated AML [195]. Clinical trials in AML have analysed vitamin C levels (NCT03526666) as well as the effect of oral vitamin C supplementation in improving the response to DNA methyltransferase inhibitors via increasing DNA demethylation (NCT0287727).

### 4.7. Targeting Mutated Metabolic Pathways

An example of precision medicine in cancer metabolism is provided by drugs that target mutant isocitrate dehydrogenase (IDH), recently introduced in AML treatment. IDH inhibitors are very encouraging drugs for AML patients with IDH mutations, especially for the elderly and R/R. Ivosidenib (IDH1 inhibitor) showed significant clinical benefit with azacitidine as compared with a placebo and azacitidine in IDH1 AML patients ineligible for intensive induction chemotherapy [196]. Enasidenib (IDH2 inhibitor) showed a significant increase in the survival rate in R/R AML IDH2 patients not eligible for HSCT [197], and, in a phase 3 trial, enasidenib displayed improved event-free survival compared to a conventional care regimen [198].

## 5. Impact of Customised Nutritional, Behavioural and Physical Interventions on Leukaemic Metabolism

**Figure 3:**
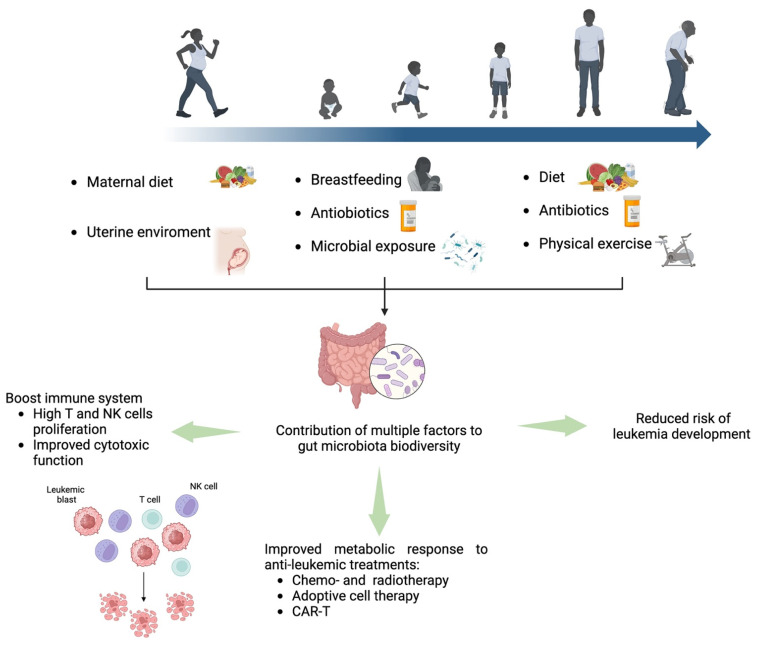
Impact of nutritional, behavioural and physical approaches on gut microbiota diversity, mediating immune responses and improving the metabolic response to anti-leukaemic treatment. Created in BioRender. Tettamanti, S. (2025) https://BioRender.com/p91b288 (accessed on 18 December 2024).

Increasing attention has been focused on nutrition in cancer metabolism. Several studies suggest that both malnutrition and obesity are associated with adverse outcomes in leukaemia [199]. After uterine life, the maternal diet during gestation affects the offspring, with evidence showing that the maternal intake of fruits and vegetables is associated with a reduction in the risk of developing leukaemia in newborns [200,201,202], likely due to the action of folic acid and other vitamins [203]. Moreover, breastfeeding has been documented to be protective against paediatric leukaemia and lymphoma occurrence, thanks to its positive action on the immune system’s status and performance [204,205]. To achieve balanced immune responses over a lifetime, the priming of the immune system is required. Evidence suggests that a deficit in microbial exposure during the first year of life is linked to an abnormal immune response to infections in later childhood. This altered response may contribute to the progression of pre-leukaemic clones, formed in utero, into clinical ALL—a transformation that occurs in approximately 1% of pre-leukaemic clones [206]. This concept, which was first predicted as an immunological theory [207], has been confirmed in case/control epidemiological studies and meta-analyses on the collected clinical data [208,209]. Independently of perinatal imprinting, studies show how diet has an impact on both the development and treatment of leukaemia [210], and specific nutritional regimens have been proposed as complementary metabolic strategies for cancer treatment and prevention. For example, the ketogenic diet was found to be beneficial against cancer through a reduction in glucose levels and impairment in glycolytic metabolism, which does not allow the processing of ketone molecules as it occurs in normal cells [211]. Preclinical studies support starving as an approach to successfully treat leukaemia [212,213]. Further details on dietary approaches for cancer treatment can be found in a recent review by Golonko et al. [213]. The microbiota plays a potential role in linking various exposure factors, such as breastfeeding, microbial exposure and diet, with the leukaemia risk [210] (Figure 3). Clear evidence shows that the setting up of the gut microbiome in the first years of life [214,215] has relevant effects on both metabolism and immunity [216,217]. Moreover, antibiotic treatment in oncological disease is associated with poorer clinical outcomes, with one of the responsible mechanisms being the antibiotic-induced alteration of the gut microbial flora [218]. Patients with leukaemia are characterised by intestinal dysbiosis already at the onset of the disease when compared to healthy subjects [219]. A two-sample Mendelian randomisation study described a causal relationship between the gut microbiota and leukaemia, identifying potential pathogenic bacteria and probiotic taxa associated with disease onset [220]. Studies in ALL report a reduction in *Firmicutes*, responsible for butyrate production [221], a metabolite with anticancer activity that is implicated in intestinal barrier damage repair [222,223]. Intestinal barrier damage, described also in AML, accelerates lipopolysaccharide (LPS) leakage into the bloodstream, and this is associated with disease progression [223]. Notably, the microbiome plays a role in regulating the immune system’s antitumour responses to different types of therapeutic strategies, such as chemotherapy [224], radiotherapy [225,226], immune checkpoint inhibition [227], adoptive cell therapy [228] and CAR T cell therapy [229,230]. A multicentre longitudinal study in patients undergoing HSCT described how the higher diversity of the intestinal microbiome was associated with lower graft vs. host disease (GVHD)-related mortality [231]. Finally, growing evidence suggests the benefit of physical exercise in children and adolescents with leukaemia at the level of both improving cellular metabolism and immune responses. Physical exercise significantly reduces the risk of cancer development and has been shown to be beneficial also for patients’ responses to therapy [232,233,234]. The feasibility, safety and efficacy of a motor intervention in onco-haematological patients was described in different treatment phases [235], with cooperative action to obtain successful treatment, such as monoclonal Ab (antibodies) [236] and chemotherapy [237]. Immune stimulation via the modulation of oxidative stress and inflammation is one of the beneficial effects of exercise regarding cancer prevention and treatment [237,238], associated with a reduction in immune senescence [239,240]. Exercise not only works as an adjuvant to improve T-cell fitness, reducing senescence and exhaustion [240], but also improves human NK cells’ ability to eliminate multiple myeloma and lymphoma cell lines [241]. Exercise may also have a role in modulating the gut microbiota [240]. Increased microbiota diversity has been described in exercised rats [242], and favourable changes in the human microbial flora were observed following a 6-week exercise intervention program, which were reversed with sedentary lifestyles [243]. As reported in this section, in the past few years, several nutritional, behavioural and physical approaches have emerged to align with conventional therapy, exploiting the metabolism to strengthen leukaemia control as well as to improve patients’ quality of life. Moreover, the influence of lifestyle factors on immune modulation, such as diet and exercise, highlights the need for a comprehensive approach that encompasses both the prevention and treatment of acute leukaemia.

## 6. Discussion

With the advent of single-cell technologies, the possibility to untangle the intratumour heterogeneity and the role of the tumour microenvironment in cancer initiation and progression is greatly enhanced, allowing for unprecedented insights into the complexity and dynamic interactions within tumours. By applying and integrating different single-cell technologies, we can simultaneously identify subclonal populations with distinct mutations, study how tumours respond to signals from surrounding stromal and immune cells, investigate the metabolic dependencies of leukaemic cells and identify possible ways to target them. This has allowed the clinical development of molecules targeting metabolism at different levels to impair leukaemic cell survival and improve leukaemia treatment. The long-term challenge is to develop personalised interventions tailored to individual patients based on their unique genetic, immunological, metabolic and microbiota profiles to achieve optimal treatment outcomes, possibly with the addition of specific nutritional, behavioural and physical approaches. While the benefits of such analyses are tangible, several challenges need to be addressed to fully harness their potential and impact clinical decision-making. Firstly, integrating data at a single-cell resolution and for each patient is challenging due to the difficulties in obtaining standardised protocols, the high costs of the analyses and the need for interpretability in a short timeframe. Another critical challenge is related to the specificity of metabolic treatment interventions. Disrupting metabolism can impair cancer cell growth and enhance the treatment efficacy; however, healthy cells may also express the targeted metabolic pathways, leading to unsolicited effects. For example, healthy proliferative tissue in humans also depends on nucleotide synthesis pathways, and treatment with nucleotide inhibitors such as methotrexate or 6-mercaptopurine commonly causes bone marrow suppression, mucositis and hair loss. Therefore, achieving the selective targeting of specific pathways that are upregulated only in tumour cells is crucial to minimise damage to healthy cells. Along with side effects, another challenge is represented by the ability of cancer cells to rewire metabolic pathways when exposed to precision therapies, leading to resistance, as shown, for example, by FLT3 leukaemia-initiating cells (LICs) in AML when exposed to venetoclax–azacitidine treatment [244], which increased FAO to maintain OXPHOS upon amino acid loss. Resistance can be overcome by using combination therapies that simultaneously inhibit multiple pathways [244,245]. Incorporating dietary and physical interventions into leukaemia treatment also presents significant challenges, especially for paediatric patients. Studies indicate that adherence to nutritional guidelines is often delayed by various factors, including personnel-related issues (such as a lack of awareness, scepticism about outcomes or a lack of consensus), guideline-related limitations and external barriers (e.g., poor collaboration) [246]. Patient adherence to strict diets, which may involve fasting periods, is also difficult to maintain, and robust evidence is needed to support the integration of tailored dietary plans into leukaemia care. Similar challenges arise with physical training programs, as these can be demanding for vulnerable patients undergoing intensive chemotherapy cycles.

## 7. Conclusions

Single-cell technologies have revolutionised our understanding of cancer by allowing us to study tumour heterogeneity, interactions within the tumour microenvironment and metabolic dependencies in leukaemia. These advances have led to the development of targeted therapies that aim to disrupt metabolic pathways that are critical for leukaemic cell survival. However, challenges remain, such as integrating complex single-cell data into clinical decision-making, ensuring the specificity of metabolic interventions to avoid side effects and overcoming resistance mechanisms in leukaemic cells. Additionally, incorporating dietary and physical interventions, especially for paediatric patients, presents practical difficulties. Ultimately, a “micro-to-macro” approach, combining molecular data with “macro” factors, such as nutritional, physical and behavioural characteristics, will pave the way for more effective, tailored treatments for leukaemia.

## Figures and Tables

**Table 1:** Comparative table of methods used to study intratumour heterogeneity.

Method	scDNAseq	scRNAseq	CyTOF	MS	Seahorse	SCENITH
Output	Mutational status	Transcriptomic profile	Protein expression	Proteomic and metabolomic profile	Metabolic capacities and dependencies	Metabolic capacities and dependencies
Cell purification required	No *	No *	No	Yes	Yes	No
Single-cell resolution	Yes	Yes	Yes	Yes	No	Yes
Compatible with cell sorting	Yes	Yes	No	No	No	Yes
Readout	Gene mutations and protein expression	Gene expression and protein expression	Protein expression	Metabolite levels	Changes in extracellular pH and [O_2_]	Changes in protein synthesis levels
Targeted	Yes	No	Yes	No	n.a.	Yes
Cost per assay (USD)	++++	+++	++	+++	++	++

* Cell purification might be suggested to focus the sequencing on populations of interest and therefore reduce the sequencing cost.

**Table 2:** Overview of clinically approved drugs with metabolic effects in the treatment of acute leukaemia.

Metabolic Action	Compound	Mechanism of Action	Disease	FDA-Approval/Clinical Trial
Amino Acid	L-Asparaginase	Asparagine and glutamine depletion	ALL	FDA-approved
Nucleotide	6-Mercaptopurine	Inhibits purine synthesis by inhibiting PPAT	ALL	FDA-approved
Nucleotide	Methotrexate	Inhibits thymidine synthesis by inhibiting folic acid metabolism (competitive inhibitor of DHFR)	ALL, AML	FDA-approved
Nucleotide	5-Fluorouracil	Blocks thymidine synthesis by inhibiting TS	ALL	FDA-approved
Nucleotide	6-Thioguanine	Guanine analogue, inhibits nucleotide biosynthesis	ALL, AML	FDA-approved
Nucleotide	Cytarabine	Pyrimidine analogue, inhibits nucleotide biosynthesis	ALL, AML	FDA-approved
Nucleotide	Nelarabine	Purine analogue, inhibits nucleotide biosynthesis	T-ALL	FDA-approved
Nucleotide	Gemcitabine	Pyrimidine analogue, inhibits nucleotide biosynthesis	ALL, AML	FDA-approved

Acute lymphoblastic leukaemia (ALL); acute myeloid leukaemia (AML); phosphoribosyl pyrophosphate aminotransferase (PPAT); thymidylate synthase (TS).

**Table 3:** Overview of ongoing clinical trials on metabolic tailored therapies in acute leukaemia.

Metabolic Action	Compound	Mechanism of Action	Disease	Clinical Trial	Notes/Results
Mitochondrial	Tigecycline	Inhibits ETC synthesis	R/R AML	NCT01332786 phase 1	Tigecycline monotherapy in R/R AML, not suitable for conventional chemotherapy
Mitochondrial	Devimistat, CPI-613	PDH inhibitor, TCA cycle inhibitor	AML	NCT02484391 phase 2	Devimistat with cytarabine and mitoxantrone in AML
Mitochondrial	Venetoclax	Induces release of cytC and apoptosis by structural alterations of mitochondrial membrane	AML	NCT02287233 phase 1/2 NCT02203773 phase 1/2	In combination with hypomethylating agent, shows tolerable and promising activity
Mitochondrial	Metformin	Overall: OXPHOS and TCA cycle inhibitor via activation of AMPK Inhibition of ETC complex I, mTOR, mGPD and other pathways: immunomodulatory and anti-aging	Childhood ALL	NCT01324180 phase 1 [166]	Metformin combined with chemotherapy (VPLD) In relapsed childhood ALL, revealed positive clinical effects
			ALL	NCT03118128 and NCT05326984, phase 1	Metformin with chemotherapy in ALL with high levels of ABCB1 drug resistance gene
Mitochondrial	Metformin with Cytarabine		R/R AML	NCT01849276, phase 1	Metformin in combination with cytarabine, no results posted yet
Mitochondrial	Metformin with Devimistat		R/R AML	NCT05854966, phase 2	Not open yet
Amino acid	L-Asparaginase	Asparagine and glutamine depletion	AML	NCT02283190 phase 1	No results posted yet
Amino acid	BCT-100	Arginine depletion	R/R ALL, AML	NCT03455140 phase 1/2	Clinical safety
Amino acid	CB-839	Glutaminase inhibitor (glutamine depletion)	AML, ALL	NCT02071927 phase 1	Well tolerated
Fatty acids	Pravastatin	HMGCR inhibitor, rate-limiting enzyme in MVA synthesis	R/R AML	NCT00107523, NCT00840177 phase 1/2	Pravastatin with idarubicin and cytarabine
Fatty acids	Pitavastatin	HMGCR inhibitor, rate-limiting enzyme in MVA synthesis	AML	NCT04512105 phase 1	Pitavastatin with venetoclax
Antioxidant	Ascorbic acid	Increases iron availability, Promotes tet2 activity	AML	NCT0352666, NCT02877277	Observational study Randomised, double-blinded, placebo-controlled pilot study
Mutations in metabolic genes	Ivosidenib	Mutant IDH1 inhibitor	AML IDH1	NCT03173248 phase 3	Significant clinical benefit of ivosidenib with azacitidine compared to placebo + azacitidine
Mutations in metabolic genes	Enasidenib	Mutant IDH2 inhibitor	R/R AML IDH2	NCT01915498 phase 1/2	Significantly prolongs relative survival among patients with R/R AML and an IDH2 mutation ineligible for HSCT
			R/R AML IDH2	NCT02577406 phase 3	EFS was meaningfully improved with enasidenib

Acute lymphoblastic leukaemia (ALL); acute myeloid leukaemia (AML); relapse refractory (R/R); HMG-CoA reductase (HMGCR); mevalonic acid (MVA); electron transport chain (ETC); oxidative phosphorylation (OXPHOS); pyruvate dehydrogenase (PDH); tricarboxylic acid (TCA); 5-adenosine monophosphate-activated protein kinase (AMPK); mammalian target of rapamycin (mTOR); mitochondrial glycerophosphate dehydrogenase (mGPD); vincristine, dexamethasone, doxorubicin, and PEG-asparaginase (VPLD); haematopoietic stem cell transplant (HSCT); event-free survival (EFS).

## Abbreviations

2-DG	2-Deoxyglucose
5,10-mTHF	5,10-Methylenetetrahydrofolate
α-KT	Alpha-ketoglutarate
AA	Amino acids
AC	Acid ceramidase
ACLY	ATP-citrate-lyase
ALL	Acute lymphoblastic leukaemia
AML	Acute myeloid leukaemia
AMPK	5′ AMP-activated protein kinase
ASNase	Asparaginase
ASNS	Asparagine synthase
BCAA	Branched-chain amino acids
BCATs	Mitochondrial BCAA-transaminases
Bcl-2	BCL2 apoptosis regulator
BM	Bone marrow
CRES6	Ceramide synthase 6
CITE-seq	Cellular Indexing of Transcriptomes and Epitopes by Sequencing
CyTOF	Cytometry by time-of-flight
CML	Chronic myeloid leukaemia
CNV	Copy number variation
CPT1a	Carnitine palmitoyl transferase 1a
CT2	Carnitine transporter 2
D2HG	D-2-hydroxyglutarate
DHFHR	Dihydrofolate reductase
ECAR	Extracellular acidification rate
EFS	Event-free survival
ETC	Electron transport chain
FA	Fatty acids
FAO	Fatty acid oxidation
*FASN*	Fatty acid synthase gene
FIS1	Mitochondrial fission protein 1
GATase	Q-amido-transferase
GCS	Glycine cleavage complex
Gln	Glutamine
GLS	Glutaminase
GLUD1	Glutamate dehydrogenase
GSH	Glutathione
GVHD	Graft vs. host disease
HK2	Hexokinase 2
HMG-CoA	3-Hydroxy-3-methylglutaryl-coenzyme A
HSCs	Haematopoietic stem cells
HSCT	Haematopoietic stem cell transplant
HSPC	Haematopoietic stem and progenitor cells
IMC	Imaging mass cytometry
IDH 1/2-	Isocitrate dehydrogenase 1/2
IDO	Indoleamine 2,3 dioxygenase
KAT7	Lysine acetyltransferase 7
KYN	Kynurenine
LAESI-MS	Laser ablation electrospray ionisation mass spectrometry
LDHB	Lactate dehydrogenase b
LIS	Leukaemia-initiating cells
LPS	Lipopolysaccharide
LSCs	Leukaemic stem cells
LSC-MS	Live single-cell mass spectrometry
MALDI-MS	Matrix-assisted laser desorption/ionisation mass spectrometry
MAT	Methionine-adenosyl-transferase
MCL1	Induced myeloid leukaemia cell differentiation protein
MCU	Mitochondrial calcium uniporter
Met	Methionine
mGDP	Mitochondrial glycerophosphate dehydrogenase
mitoROS	Mitochondrial ROS
MRD	Minimal residual disease
MS	Mass spectrometry
MTA	5′-Methylthioadenosine
MTCH2	Pyruvate mitochondrial carrier
mtDNA	Mitochondrial DNA
mTOR	Mammalian target of rapamycin
MVA	Mevalonic acid
NEEA	Non-essential amino acids
NMR	Nuclear magnetic resonance
OCR	Oxygen consumption
ODC	Ornithine-decarboxylase
OXPHOS	Oxidative phosphorylation
PAPi	Pathway activity profiling
PDH	Pyruvate dehydrogenase
PER	Proton efflux rate
PHGDH	Phosphoglycerate dehydrogenase
PPP	Pentose phosphate pathway
PSAT1	Phosphoserine aminotransferase 1
PSPH	Phosphoserine phosphatase
PS	Protein synthesis
R/R	Relapsed/refractory
ROS	Reactive oxygen species
RP2D	Recommended phase 2 dose
SAH	S-adenosyl-homocysteine
SAM	S-adenosyl-methionine
SCENITH	Single-Cell Energetic Metabolism by Profiling Translation Inhibition
SCD1	Stearoyl CoA desaturase 1
scDNA-Seq	Single-cell DNA sequencing
scMEP	Metabolic regulome of single cells
scMS	Single-cell mass spectrometry
SCP	Single-cell proteomics
scRNA-Seq	Single-cell RNA sequencing
SHMT	Serine hydroxy methyltransferase
SNV	Single-nucleotide variation
SP1	Sphingosine 1 phosphate
SSP	Serine synthesis pathway
SRC	Spare respiratory capacity
TCA	Tricarboxylic acid
TDO	Tryptophan-2,3-dioxygenase
TET	Tet methyl cytosine dioxygenase
Trp	Trypthophan
TS	Thymidylate synthase
VDAC1	Voltage-dependent anion channel 1
VPLD	Vincristine, dexamethasone, doxorubicin and PEG-asparaginase
WES	Whole-exome sequencing
